# Right ventricular free wall dyskinesis in the setting of left ventricular non compaction: a case report

**DOI:** 10.1186/1756-0500-7-787

**Published:** 2014-11-05

**Authors:** Tamara Glavinovic, David YC Cheung, Francisco J Cordova Perez, Anita Soni, Brett Memauri, Davinder S Jassal

**Affiliations:** Section of Internal Medicine, Department of Internal Medicine, Faculty of Medicine, University of Manitoba, Winnipeg, Manitoba Canada; Institute of Cardiovascular Sciences, St. Boniface Research Centre, University of Manitoba, Winnipeg, Manitoba Canada; Section of Cardiology, Department of Internal Medicine, Faculty of Medicine, University of Manitoba, Winnipeg, Manitoba Canada; Department of Radiology, Faculty of Medicine, University of Manitoba, Winnipeg, Manitoba Canada; Bergen Cardiac Care Centre Section of Cardiology, Department of Internal Medicine, St. Boniface General Hospital, 409 Taché Avenue, Winnipeg, Manitoba R2H 2A6204-237-2023 Canada

**Keywords:** Left ventricular non compaction, Multi-modality cardiac imaging

## Abstract

**Background:**

Left ventricular non compaction is a relatively rare congenital disorder characterized by prominent trabeculations and intertrabecular recesses with the potential for thromboembolism, arrhythmias, and sudden cardiac death as adverse effects. Echocardiography has traditionally been employed as the primary mode of imaging; however, with the advent of cardiac magnetic resonance as a more precise imaging technique, the disorder known as left ventricle non compaction is becoming more broadly defined with increasing recognition of right ventricle (RV) involvement.

**Case presentation:**

This report describes a 52-year-old Caucasian female with new onset atrial fibrillation with an unusual finding of left ventricular non compaction and right ventricular dysfunction on transthoracic echocardiogram with preserved left ventricular ejection fraction. Cardiac magnetic resonance imaging demonstrated a disproportionately affected right ventricle, with apical free wall dyskinesis.

**Conclusions:**

This case illustrates the unique occurrence of left ventricular non compaction with preserved ejection fraction alongside RV free wall dyskinesis and RV systolic dysfunction. The significance of this is yet unknown given the paucity of existing literature. This report serves to highlight the vast heterogeneity within left ventricular non compaction as we are better able to delineate this disorder using increasingly sophisticated imaging techniques.

## Background

Left ventricular non-compaction (LVNC) is a relatively rare congenital disorder thought to be either genetically familial or sporadic [1]. The estimated incidence of LVNC in the general population is between 0.05-0.25% per year [2], whereas its prevalence remains unknown as many patients are asymptomatic and thus, never diagnosed. The characteristic features of LVNC are prominent trabeculations and inter-trabecular recesses within the left ventricle (LV). The etiology of LVNC is presumed to be due to arrest during embryogenesis. Under normal circumstances, compaction of the myocardium and disappearance of trabeculations and the associated deep recesses occur between the 5^th^ and 8^th^ week of fetal development [3]. LVNC is typically defined in the absence of other congenital or acquired cardiomyopathies. The clinically adverse events seen in LVNC are thromboembolism, ventricular arrhythmias, congestive heart failure with both systolic and diastolic dysfunction and lastly, sudden cardiac death. Echocardiography has been the primary imaging modality used to delineate the presence of LVNC, whereas cardiac magnetic resonance imaging (CMR) is now being acknowledged as a more precise method of evaluating this disorder. CMR is of benefit with regards to measurement of myocardial thickness between compacted and non-compacted regions [4]. CMR is also helpful in determining the function of both right and left ventricles in a manner more precise than echocardiography. While LVNC was traditionally thought to affect only the LV, it is now known that there exists a spectrum of involvement of the right ventricle (RV).

## Case presentation

**Figure 1:**
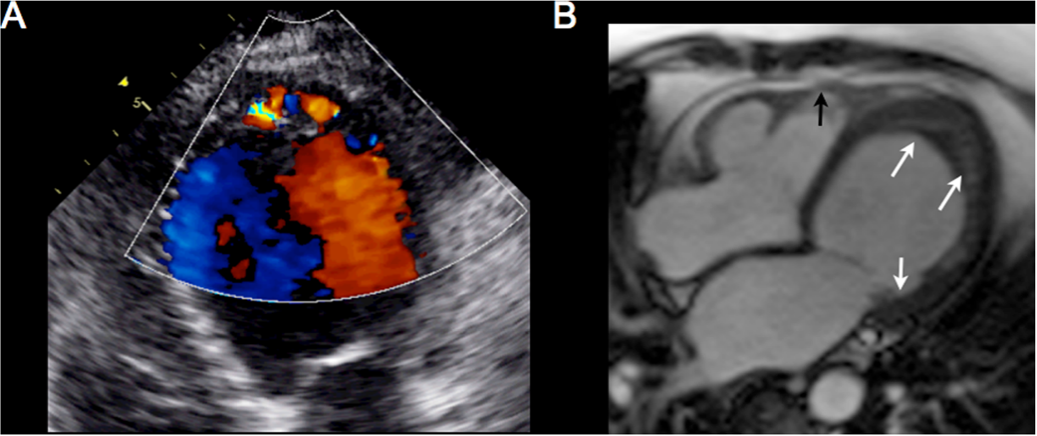
**Multimodality cardiac imaging of left ventricular non compaction. A)** An apical 4 chamber view on transthoracic echocardiography demonstrating trabeculations and recesses within the left ventricular apex on color Doppler consistent with left ventricular non compaction. **B)** A horizontal long axis steady-state free precession view on cardiac magnetic resonance imaging confirming significant hypertrabeculations involving the left ventricualr apex, lateral wall, and extension into the posterior mitral valve leaflet (white arrows). There is also evidence of dyskinesis of the right ventricular free wall that is mildly aneurysmal (black arrows).

A 52-year-old Caucasian female with new onset atrial fibrillation and a family history of premature sudden death underwent transthoracic echocardiography (TTE) to evaluate the presence of structural heart disease. A 12 lead baseline EKG demonstrated no evidence of an Epsilon wave nor repolarization abnormalities and there was no evidence of premature ventricular contractions nor ventricular tachycardia on 48 hour Holtor monitoring. TTE demonstrated abnormal thickening of the left ventricular (LV) apex with trabeculations and recesses, consistent with LVNC (Figure 1A). The LV ejection fraction on TTE was >60%. Although the anterior mitral valve was structurally normal, the posterior mitral valve leaflet (PMVL) was thickened and embedded within the lateral trabeculated LV myocardium, resulting in severe mitral regurgitation. There was no evidence of pulmonary hypertension as the pulmonary artery systolic pressure was calculated at 30 mm Hg. CMR imaging confirmed the atypical appearance of LVNC extending from the LV apex to the basal portion of the LV with significant restriction of the PMVL (Figure 1B: white arrows). Additionally, on CMR, although the RV cavity was not dilated, there was dyskinesis of the apical free wall of the RV with a RVEF calculated at 43% (Figure 1B: black arrow). This appeared to be consistent with an atypical variant of LVNC. The patient was subsequently discharged home and is undergoing evaluation for implantable cardioverter defibrillator placement.

## Discussion

This case illustrates the unique occurrence of LVNC with preserved ejection fraction alongside RV free wall dyskinesis and RV systolic dysfunction. Although it is plausible that the RV abnormalities identified on CMR may be associated with another underlying diagnosis of arrythmogenic RV dysplasia, there were no other clinical or electrocardiographic features supportive of this diagnosis. While the significance of RV involvement in LVNC is yet unknown given the paucity of existing literature, this report serves to highlight the vast heterogeneity within LVNC as we are better able to delineate this disorder using increasingly sophisticated imaging techniques. Several recent studies have demonstrated RV involvement, though focus has been on concomitant LV systolic dysfunction: a study of 56 patients looked at right ventricular involvement in LVNC, and focused specifically on the presence of RV systolic dysfunction, RV non-compaction, and myocardial fibrosis. It was found that RV systolic dysfunction was present in 16% of patients; however, all had associated LV systolic dysfunction [5]. A prior study by *Leung et al*. demonstrated RVEF <35% in half of patients, and these similarly had lower LV ejection fractions [6]. Similarly, *Stacey et al*. noted that those patients with reduced RV ejection fractions were at higher risk for adverse outcomes, including congestive heart failure, thromboembolism, and arrhythmias, even after having adjusted for LVEF [7]. A study looking at patients in Sub Saharan Africa found higher rates of RV non-compaction than previously reported. Using TTE exclusively, they identified 61% of patients as having RV dysfunction, though there was a high rate of concomitant pulmonary hypertension, tricuspid regurgitation and LV dysfunction, thus the ability to determine that RV dysfunction could occur in isolation was not possible [8]. These findings may suggest that systolic dysfunction seen in both ventricles is a marker of an advanced disease state, yet we have yet to elucidate what risks isolated RV systolic function may confer and what this means regarding prognostication.

## Conclusion

This report serves to highlight the vast heterogeneity that seems to be expanding as we continue to increase our understanding of LVNC in an era of more sophisticated imaging techniques including CMR.

## Consent

Written informed consent was obtained from the patient for publication of this Case report and any accompanying images. A copy of the written consent is available for review by the Editor-in-Chief of this journal.

## Abbreviations

CMR: Cardiac magnetic resonance imaging
LV: Left ventricle
LVEF: Left ventricular ejection fraction
LVNC: Left ventricular non compaction
PMVL: Posterior mitral valve leaflet
RV: Right ventricle
TTE: Transthoracic echocardiography.
